# Introduction of non-linear elasticity models for characterization of shape and deformation statistics: application to contractility assessment of isolated adult cardiocytes

**DOI:** 10.1186/2046-1682-4-17

**Published:** 2011-08-22

**Authors:** Carlos Bazan, Trevor Hawkins, David Torres-Barba, Peter Blomgren, Paul Paolini

**Affiliations:** 1 Computational Science Research Center, San Diego State University, 5500 Campanile Drive, San Diego, CA 92182-1245, USA; 2 Department of Mathematics & Statistics, San Diego State University, 5500 Campanile Drive, San Diego, CA 92182-7720, USA; 3 Department of Biology, San Diego State University, 5500 Campanile Drive, San Diego, CA 92182-4614, USA

## Abstract

**Background:**

We are exploring the viability of a novel approach to cardiocyte contractility assessment based on biomechanical properties of the cardiac cells, energy conservation principles, and information content measures. We define our measure of cell contraction as being the distance between the shapes of the contracting cell, assessed by the minimum total energy of the domain deformation (warping) of one cell shape into another. To guarantee a meaningful *vis-à-vis *correspondence between the two shapes, we employ both a data fidelity term and a regularization term. The data fidelity term is based on nonlinear features of the shapes while the regularization term enforces the compatibility between the shape deformations and that of a hyper-elastic material.

**Results:**

We tested the proposed approach by assessing the contractile responses in isolated adult rat cardiocytes and contrasted these measurements against two different methods for contractility assessment in the literature. Our results show good qualitative and quantitative agreements with these methods as far as frequency, pacing, and overall behavior of the contractions are concerned.

**Conclusions:**

We hypothesize that the proposed methodology, once appropriately developed and customized, can provide a framework for computational cardiac cell biomechanics that can be used to integrate both theory and experiment. For example, besides giving a good assessment of contractile response of the cardiocyte, since the excitation process of the cell is a closed system, this methodology can be employed in an attempt to infer statistically significant model parameters for the constitutive equations of the cardiocytes.

## Background

### Introduction

Cardiovascular research based on enzymatically dissociated cardiocytes has been fundamental for the discovery of the mechanisms that govern the heart. The use of the cardiocyte as the basis for cardiac functionality has provided some of the most revealing information regarding heart function. Among the many findings, it has revealed the crucial molecular changes that occur during pathological conditions of the heart. The details regarding the excitation-contraction coupling, calcium transient signal (movement of the calcium ion Ca^2+^), gene and protein expression, and contractility are all important mechanisms and functions that can be readily studied in the isolated cardiocytes at all stages of development and they are routinely performed during research studies [1-6].

Contractility in adult cardiocytes is commonly interpreted as the ability of the cardiac cell to generate force and to shorten. Some of the different methodologies devised to study the contractile process include laser diffraction [7], photodiode arrays [8], scanning ion conductance microscopy [6], and those employing microscopic cell image analysis [9-12]. Historically the most widely used methods have been those involving cell image analysis, although all the methods show some positive and negative characteristics that are worthy of attention.

Methods such as the scanning ion conductance microscopy require elaborate and expensive equipment [6]. This technique, combined with laser confocal microscopy, is one of the few methods that has been capable of providing a measure of cardiocyte height during contraction. Other methods, such as light diffraction techniques, have been applied to the study of muscle mechanics since the nineteenth century with relatively high reliability. Nonetheless, they are very dependent upon several factors including the temporal resolution of the detection system and optical artifacts [2]. The sarcomere striation pattern analysis method has also been used as a way to quantify contractility. This technique can achieve high temporal resolution with the aid of charge-coupled device line array detectors and it provides a measure of individual sarcomere lengths along the cell [13,14]. A drawback of this method is its vulnerability to errors introduced by slight rotational and translational changes that normally occur during cell contraction [2].

Although the image analysis methods have been widely used with relative high reliability, the results they provide are often prone to the introduction of error due to the aforementioned rotation or vertical and horizontal displacement of the cardiocyte during contraction. Our proposed approach aims to provide a cardiocyte contraction analysis method that successfully captures the full extent of the contractile behavior, while minimizing the need for elaborate equipment and the effects that the cardiocyte's movements have on the acquired signal.

### Previous Work

**Figure 1:**
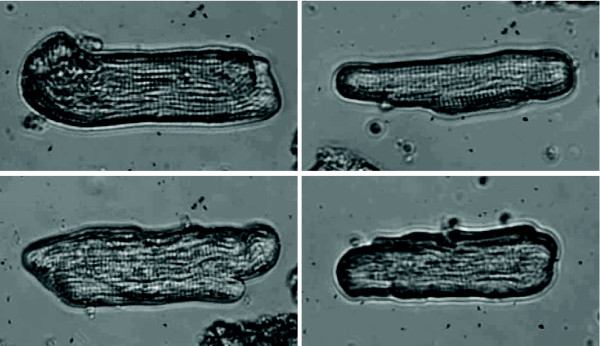
**Frames from contracting cardiocytes**. Frames extracted from videos of adult cardiocytes depicting contractions. These are typical rod-shaped cardiocytes isolated from an adult mammalian heart.

A widely used video-based method to measure contraction in adult cardiocytes involves a device capable of capturing the extent and rate of length shortening between the cell's ends, the so-called *edge detection method *[15-17,11]. In this technique, a single video raster line oriented along the longitudinal axis of the cell shows high contrast at the cell boundaries. These serve as tracking points and their separation distance corresponds to cell length. The method generally produces satisfactory results and has been a broadly used approach for measuring contractile responses of adult cardiocytes for over twenty years [2,16]. Some practical difficulties have been identified during the implementation of the edge detection method for measuring adult cardiocyte contractility [2]. The changes in cardiocyte geometry, dynamic torquing, and rotation can lead to errors in the measurement [2,15,16,5]. Figure 1 shows frames extracted from videos of adult cardiocytes depicting contractions.

In a previous communication, Bazan, Torres-Barba, Paolini and Blomgren [18] described a computational pipeline for the comprehensive assessment of contractile responses of enzymatically dissociated adult cardiac myocytes. The methodology comprises the following stages: digital video recording of the contracting cell, edge preserving total variation-based image smoothing, segmentation of the smoothed images, contour extraction from the segmented images, shape representation by Fourier descriptors, and contractility assessment. The physiologic application of the methodology was evaluated by assessing the overall contraction in isolated adult rat cardiocytes. The results demonstrated the effectiveness of the approach in characterizing the more appropriate, two-dimensional, shortening in the contraction process of adult cardiocytes. The authors in [18] compared the performance of their method to that of the aforementioned edge detection system. The method not only provided a more comprehensive assessment of the myocyte contraction process, but can potentially eliminate the historical concerns and sources of errors caused by myocyte rotation, bending, or translation during contraction [2,9,19].

In this paper, we are exploring the viability of a novel approach to cardiocyte contractility based on biomechanical properties of the cardiac cells, energy conservation principles, and information content measures. The proposed methodology was inspired by the works of Byung-Woo Hong *et al*. [20-22], School of Computer Science and Engineering, Chung-Ang University, Seoul, Korea; and Andrew D. McCulloch *et al*. [23-26], Department of Bioengineering, University of California San Diego, La Jolla, California.

## Methods

### Cardiocyte Shape Representation With Integral Kernels

**Figure 2:**
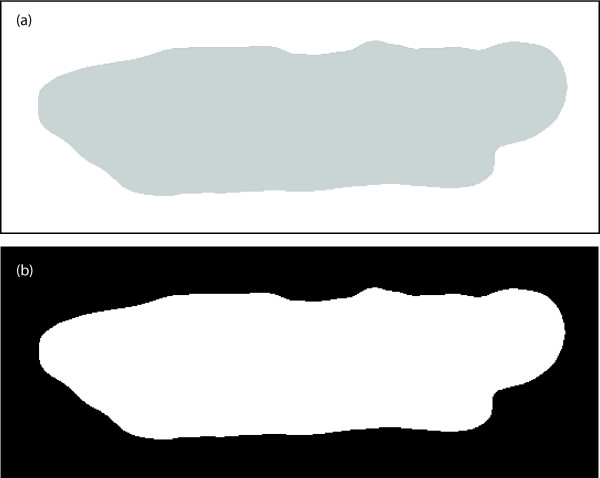
**Cardiocyte shape representation**. (a) Representation of the cardiocyte shape by a closed planar region, *D*. (b) Binary representation *S *of the cardiocyte shape.

We wish to retrieve the information embedded in the shapes of a contracting cardiocyte like the ones shown in Figure 1. In other words, we want to analyze closed planar regions D ⊂ ℝ^2^, and their boundaries (finite perimeters), as depicted in Figure 2(a). We will describe these regions by binary images, Figure 2(b), composed with a suitable class of (continuous and invertible) image domain transformation. The binary images will be represented by a characteristic function

(1) $$S\left(x\right)=S_{D}\left(x\right)=\left\{\begin{array}{cc} 1\; & \text{if}\;x\in D\\ 0\; & \text{if}\;x\notin D, \end{array}\right)$$

defined for *x *∈ Ω ⊂ ℝ^2^, with *D *⊂ Ω, where Ω is the rectangular image domain.

We will define the multi-scale nonlinear features *R_σ_*, as the convolution of the shape *S *with a family of kernels *K_σ_*, indexed by a scale *σ*. More specifically, *σ *∈ ℝ^+^, *K *: ℝ^2 ^× ℝ^+ ^→ ℝ^+^; (*x*, *σ*) ↦*K_σ _*(*x*). For convenience, we will consider the isotropic Gaussian kernel of the form

(2) $$K_{\sigma }\left(x\right)=\frac{1}{\sigma \sqrt{2\pi }}\exp\left(-\frac{|x{|}^{2}}{2\sigma^{2}}\right).$$

We will work with the non-linear features proposed by Hong, Soatto and Vese [20], which were designed to retain boundary information. They are given by one of the following two expressions,

(3) $$\begin{array}{c} R_{\sigma }:L^{1}\left(\Omega \right)\to L^{1}\left({\mathbb{R}}^{2}\right),\\ S\left(x\right)\mapsto R_{\sigma }\left(x|S\right)\overset{{}^{\circ}}{=}S\left(x\right)\left(K_{\sigma }*\left(1-S\left(x\right)\right)\right), \end{array}$$

or the symmetrized version

(4) $$\begin{array}{c} R_{\sigma }:L^{1}\left(\Omega \right)\to L^{1}\left({\mathbb{R}}^{2}\right),\\ S\left(x\right)\mapsto R_{\sigma }\left(x\left|S\right)\right)≐S\left(x\right)\left(K_{\sigma }*\left(1-S\left(x\right)\right)\right)\\ \;\;\;\;\;\;\;\;+\left(1-S\left(x\right)\right)\left(K_{\sigma }*S\left(x\right)\right). \end{array}$$

**Figure 3:**
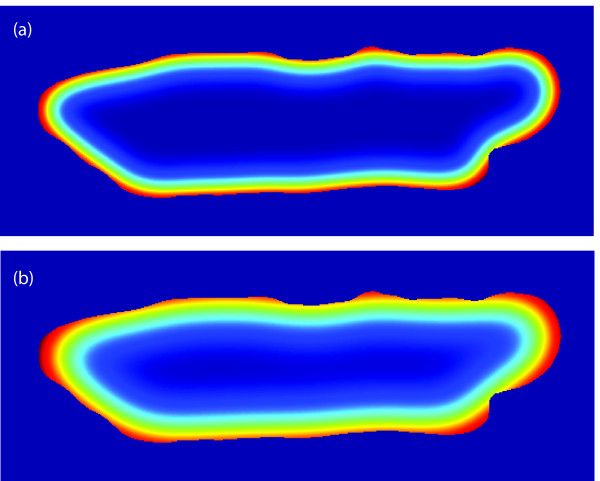
**Cardiocyte shape features (asymmetric)**. Examples of the shape features *R_σ _*(*x *| *S*) = *S *(*x*) (K_σ _* (1 - *S *(*x*))) for (a) *σ *= 15 and (b) *σ *= 25.

**Figure 4:**
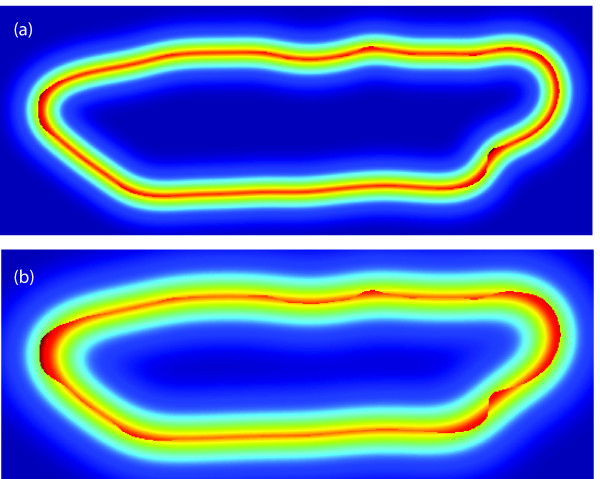
**Cardiocyte shape features (symmetric)**. Examples of the shape features *R_σ _*(*x *| *S*) = *S *(*x*) (K_σ _* (1 - *S *(*x*))) + (1 - *S *(*x*)) (K_σ _* *S *(*x*)) for (a) *σ *= 15 and (b) *σ *= 25.

The shape representation for different scales using the first (simpler) features, Eq. (3), are shown in Figure 3. The shape representation for different scales using the second (symmetric) features, Eq. (4), are shown in Figure 4. Both the binary representation (*S*) and the nonlinear shape features (*R_σ_*) include the original boundary information. However, the nonlinear shape features also encode the local shape information (up to a scale *σ*), which is not explicitly available in the binary representation.

These shape features have several useful properties (for more details on these properties, please see [20]): (i) they are very robust under the presence of noise that gets incorporated in the segmentation process; (ii) since their values depend on the local geometry, these features propagate the shape information inside and outside the boundary; (iii) because the value of the features at a point is a local statistic of the shape in a neighborhood of that point, these features capture the context of the particular shape; and (iv) these shape features are very straightforward to compute.

### Cardiocyte Contractility Assessment via Shape Matching

In this paper, the contractility analysis is done at the cellular level, thereby only measuring the overall contractions in the cell. This is consistent with the contraction measurements that are being used in our laboratory. We are working on a similar energy conservation and information content approach for assessing contractility in neonatal cardiocytes, where we will measure the fine granular changes in the image. Unlike adult cardiocytes, which are highly organized and quite similar in morphology, the neonatal cardiocyte is in the process of developing its contractile machinery. The neonatal cardiocyte is generally unable to retract its cell boundary during contraction, and noticeable changes occur only within the cell perimeter. For these reasons, it is difficult to perform contractile measurements on this cell type in a manner similar to that of the adult cardiocyte, in which changes in cell boundary are quantified during contraction. (Please see a recent article by Bazan, Torres-Barba, Paolini and Blomgren [27] for a previously developed computational framework for the quantitative assessment of contractile responses of isolated neonatal cardiac myocytes.)

**Figure 5:**
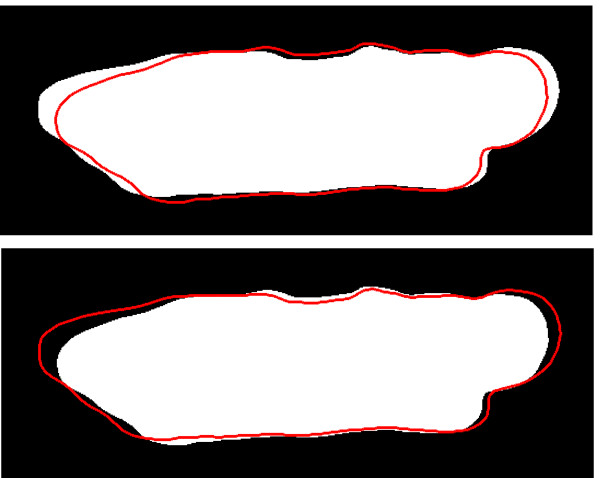
**Cardiocyte contraction/relaxation process**. Example of two shapes of the same topology that depict the contraction/relaxation process that we are trying to measure.

We are given two shapes of the same topology, *i.e*., the shapes of a relaxed and contracted cardiocyte, respectively (Figure 5), defined by the two functions *S*_1_, *S*_2 _: Ω → {0, 1}. Our intent is to transform one into the other, and *vice-versa*, in a process that resembles that of the contraction/relaxation of the cardiocyte. As proposed in [20], we will do this by *warping*, that is a domain deformation *h *: Ω → ℝ^2 ^such that *h *(Ω) = Ω and

(5) $$S_{1}\left(x\right)=S_{2}\left(h\left(x\right)\right),\;\;\forall x\in \Omega .$$

We are interested in the *distance *between the two shapes, *i.e*., our proposed measure of contraction. The distance between the shapes will be defined as the energy of the aforementioned warping. Since there are infinitely many warpings that satisfy (5), in order to make this distance unique, it is defined as the one that minimizes the energy in a suitably chosen class [28]. For instance, as proposed in [20], $d\left(S_{\text{1}},S_{\text{2}}\right)\overset{{}^{\circ}}{=}\text{mi}{\text{n}}_{h}||h||$ subject to (5), where *h *is a diffeomorphism and ||·|| is some chosen norm of integral form. This minimization process can be recast as a variational problem of the form

(6) $$d\left(S_{1},S_{2}\right)=\underset{h\in H}{\inf}\left[E_{\text{data}}\left(S_{1},S_{2}|h\right)+\alpha E_{\text{reg}}\left(h\right)\right],$$

where *H *is a suitable function space. The distance between the two shapes, as defined above, is a function of two energy components: *E*_data _(*S*_1_, *S*_2 _|*h*), which represents the data *fidelity*; and *E*_reg _(*h*), which is a *regularization *term. Both terms are explained in detail below.

In order to guarantee a meaningful *vis-à-vis *correspondence between the two shapes up to a certain scale [22] (*i.e*., a point *x *in the interior of the set that defines *S*_1 _is mapped to a point *h *(*x*) in the interior of the set that defines *S*_2_; and similarly, a point x in the exterior of the set that defines *S*_1 _is mapped to a point *h *(*x*) in the exterior of the set that defines *S*_2_), we adopt the measure of data fidelity between the two shapes *S*_1 _and *S*_2 _proposed in [20]:

(7) $$E_{\text{data}}\left(h|S_{1},S_{2}\right)=\int_{\Omega }|R_{\sigma }\left(x|S_{1}\right)-R_{\sigma }\left(h\left(x\right)|S_{2}\right){|}^{2}dx,$$

where *R_σ _*is the shape features (3) or (4).

In our application, the purpose of the regularization term *E*_reg _(*h*) is two-fold. First, it is there to render the problem well posed and is designed to penalize variations of the diffeomorphism function *h *in favor of smoothness. Second, it makes the deformations compatible with the deforming material. Several authors have provided insight into the constitutive laws that describe the mechanical responses of resting and contracting cardiac muscle, along with their regional and temporal variations [23,29-31]. The problem is, however, extremely complex and has required of elaborate combinations of multiaxial tissue testing [32,33], microstructural morphological modeling [34,35], statistical parameter estimation, and validation with measurements [36,37]. Very sophisticated numerical methods are also essential for accurate quantitative analysis in all phases of the investigations [23].

The intact cardiac muscle undergoes finite deformations during the normal cardiac cycle. Thus, the classical linear theory of elasticity is inappropriate for resting myocardial mechanics [38,39,25]. The myocardium is frequently modeled as a finite hyper-elastic material, where the second Piola-Kirchhoff stress tensor components *P_ij_*, are related to the components of the Lagrangian Green's strain tensor *E_ij_*, through the pseudo-strain energy *W*, as

(8) $$P_{ij}=\frac{1}{2}\left(\frac{\partial W}{\partial E_{ij}}+\frac{\partial W}{\partial E_{ji}}\right)-pC_{ij}^{-1},$$

where *C_ij _*is the right Cauchy-Green deformation tensor and p is a hydrostatic pressure Lagrange multiplier [23] (which we assume to be zero in this analysis). Several functional forms have been proposed for *W *[24,40,30,42,26,33]. We will adopt the transversely isotropic functional form proposed by Guccione, McCulloch and Waldman [24] that considers the fibrous structure of the myocardium. The strain energy potential W is an exponential function of the strain components *E_ij _*referred to the fiber coordinates

(9) $$\begin{array}{c} W=\frac{C}{2}\left(e^{Q}-1\right),\\ Q=b_{f}E_{11}^{2}+b_{t}E_{22}^{2}+b_{fs}\left(E_{12}^{2}+E_{21}^{2}\right), \end{array}$$

where

(10) $$E_{ij}=\frac{1}{2}\left(\frac{\partial h_{i}}{\partial x_{j}}+\frac{\partial h_{j}}{\partial x_{i}}+\frac{\partial h_{k}}{\partial x_{i}}\frac{\partial h_{k}}{\partial x_{j}}\right).$$

The aforementioned fiber coordinate system has the coordinate directions of the muscle fiber axis, the axis of the myofiber sheets, and the axis normal to the sheets, and is derived by rotating the cardiac coordinate system through the two angles that define the local myofiber-sheet orientation [24]. In Eq. (9), *E*_11 _is the fiber strain, *E*_22 _is the cross-fiber in-plane strain, and E_12 _is the shear strain in the fiber-cross fiber coordinate plane. Omens, MacKenna and McCulloch [26] have found that the material constants *C *= 1.1 kPa, *b_f _*= 9.2 kPa, *b_t _*= 2.0 kPa, and *b_fs _*= 3.7 kPa are appropriate to model the strains measured in the rat midwall. Then, the regularization term, *E*_reg _(*h*), can be written as

(11) $$E_{\text{reg}}\left(h\right)=\frac{C}{2}\int_{\Omega }\left(e^{Q}-1\right)dx.$$

The optimal correspondence given by h* is obtained by

(12) $$h^{*}=\arg\underset{h}{\min}\left(E_{\text{data}}+E_{\text{reg}}\right).$$

The energy minimization is performed in a variational framework using a gradient descent method. The Euler-Lagrange equation corresponding to the energy *E *= *E*_data _+ *E*_reg _yields the gradient direction for *h*:

(13) $$\frac{\partial h}{\partial t}=-\frac{\partial E}{\partial h}=-\frac{\partial E_{\text{data}}}{\partial h}-\alpha \frac{\partial E_{\text{reg}}}{\partial h},$$

where *α *is a small parameter (Lagrange multiplier). Using the features from Eq. (3),

*R_σ _*(*x *| *S*) = *S *(*x*) (K_σ _* (1 - *S *(*x*))), we have

(14) $$\begin{array}{ll} \frac{\partial E_{\text{data}}}{\partial h} & =\nabla S_{2}{}^{{}^{\circ}}h\cdot \{(R_{\sigma }(x|S_{1})-R_{\sigma }(h(x)|S_{2}))\\ & \cdot (K_{\sigma }*(S_{2}{}^{{}^{\circ}}h-1))\\ & +K_{\sigma }*((R_{\sigma }(x|S_{1})-R_{\sigma }(h(x)|S_{2}))\cdot S_{2}{}^{{}^{\circ}}h)\}, \end{array}$$

and, in components form, we have

(15) $$\begin{array}{ll} \frac{\partial E_{\text{reg}}}{\partial h_{1}} & =\frac{C}{2}e^{Q}\{-b_{f}\frac{\partial }{\partial x_{1}}(2u_{1}+3u_{1}^{2}+3u_{1}^{3}+v_{1}^{2}+v_{1}^{2}u_{1})\\ & -b_{t}\frac{\partial }{\partial x_{2}}(2u_{2}v_{2}+u_{2}v_{2}^{2}+u_{2}^{3})\\ & -b_{fs}\frac{\partial }{\partial x_{1}}(u_{2}^{2}+u_{2}v_{1}+u_{1}u_{2}^{2}+u_{2}v_{1}v_{2})\\ & -b_{fs}\frac{\partial }{\partial x_{2}}(u_{2}+2u_{1}u_{2}+v_{1}+v_{1}u_{1}+u_{1}^{2}u_{2}\\ & +v_{1}v_{2}+v_{1}v_{2}u_{1})\}, \end{array}$$

(16) $$\begin{array}{ll} \frac{\partial E_{\text{reg}}}{\partial h_{2}} & =\frac{C}{2}e^{Q}\{-b_{f}\frac{\partial }{\partial x_{1}}(2v_{1}u_{1}+v_{1}u_{1}^{2}+v_{1}^{3})\\ & -b_{t}\frac{\partial }{\partial x_{2}}(2v_{2}+3v_{2}^{2}+v_{2}^{3}+u_{2}^{2}+u_{2}^{2}v_{2})\\ & -b_{fs}\frac{\partial }{\partial x_{1}}(u_{2}+u_{2}v_{2}+v_{1}+2v_{1}v_{2}+u_{1}u_{2}\\ & +u_{1}u_{2}v_{2}+v_{1}v_{2}^{2})\\ & -b_{fs}\frac{\partial }{\partial x_{2}}(v_{1}u_{2}+v_{1}^{2}+v_{1}u_{1}u_{2}+v_{1}^{2}v_{2})\}, \end{array}$$

where

(17) $$\begin{array}{lll} Q & =b_{f}\frac{1}{4}{\left(2u_{1}+u_{1}^{2}+v_{1}^{2}\right)}^{2}+b_{t}\frac{1}{4}{\left(2v_{2}+v_{2}^{2}+u_{2}^{2}\right)}^{2}\; & \text{(1)}\\ & +b_{f\;s}\frac{1}{2}{\left(u_{2}+v_{1}+u_{1}u_{2}+v_{1}v_{2}\right)}^{2},\; & \text{(2)}\\ & \; & \text{(3)} \end{array}$$

With the following short-hand notation

(18) $$\begin{array}{lll} {\left(h_{1}\right)}_{x_{1}}=\frac{\partial h_{1}}{\partial x_{1}}=u_{1},\;{\left(h_{1}\right)}_{x_{2}}=\frac{\partial h_{1}}{\partial x_{2}}=u_{2}, & \; & \text{(1)}\\ {\left(h_{2}\right)}_{x_{1}}=\frac{\partial h_{2}}{\partial x_{1}}=v_{1},\;{\left(h_{2}\right)}_{x_{2}}=\frac{\partial h_{2}}{\partial x_{2}}=v_{2}. & \; & \text{(2)}\\ & \; & \text{(3)} \end{array}$$

### Average Shape of the Relaxed State

In order for us to use the energy of the warping as a measure of the cardiocyte's contractions, we need to determine a baseline that will represent the state when the cell is not contracting (relaxed). We will define this *baseline *as the average shape of the cardiocyte's relaxed state. In other words, given an ensemble of shapes {*S*_1_, *S*_2_, ..., *S_n_*}, we are interested in finding the average of the cardiocyte's shapes representing the uncontracted phase.

A video from our lab depicting a contracting cardiocyte will typically comprise about one thousand frames. On average, about ninety percent of these frames will show the cardiocyte in its relaxed state.

**Figure 6:**
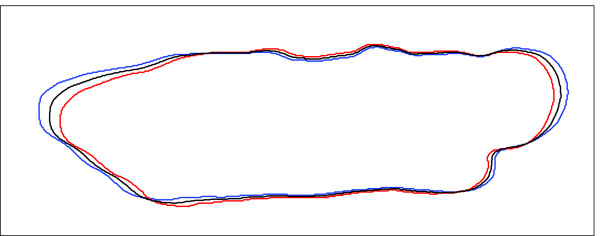
**Average relaxed shape**. The contours of a relaxed shape and a contracted shape were averaged in order to show the effectiveness of the contour averaging method. In practice, we only average the contours of uncontracted shapes.

Furthermore, the shapes of the cardiocyte in these frames are practically identical, then finding the average shape only guarantees a more unbiased measurement while providing for some regularization. Thus, in the interest of minimizing the overall computing time, we implemented a very simple averaging of contours *in lieu *of a shape averaging. The algorithm is as follows: 1) Identify the frames from each relaxed phase; 2) Obtain the contours of the shapes; 3) Resample the contours so that they will have the same number of points; 4) Find the average centroid point; 5) Interpolate the points in each contour using splines in polar coordinates; and 6) Interpolate the splines among the contours. Figure 6 shows the effectiveness of this very simple contour averaging approach where the contours of a relaxed shape and a contracted shape were averaged. We show this averaging since the average of two uncontracted contours is practically indistinguishable from the two uncontracted contours.

It is worth noting here that the aforementioned shape averaging could have been accomplished within the same shape deformation approach due to Hong, Soatto and Vese [20], where they define their shape average *M *as the shape that is closest to the ensemble, by simultaneously minimizing their energy functional equivalent to Eq. (6). In other words, they look for *M *that minimizes

(19) $$\overset{n}{\underset{i=1}{\sum }}d\left(M,S_{i}\right).$$

They perform this optimization via alternating minimization and gradient descent. Implementing the optimization process proposed in [20] was deemed to be too expensive a proposition for our purposes. Thus, we opted for performing the simpler average of contours which is two orders of magnitudes less expensive to run on a personal computer equipped with MATLAB™.

## Results and Discussion

### Experimental Results

**Figure 7:**
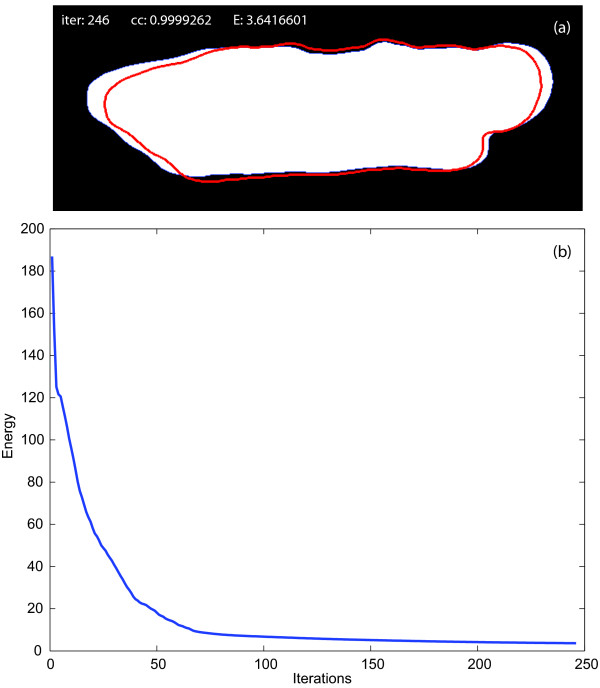
**Warping process and warping energy**. (a) Warping of the shape of a contracting myocyte into the shape of the average relaxed myocyte. After 246 iterations the template shape matched the target shape with a similarity of over 99.99% as measured by the correlation coefficient between the warped shape and the average shape. (b) Energy of the warping of the shape of a contracting myocyte into the shape of the average relaxed myocyte. The energy minimization process reaches a stable minimum after 246 iterations.

**Figure 8:**
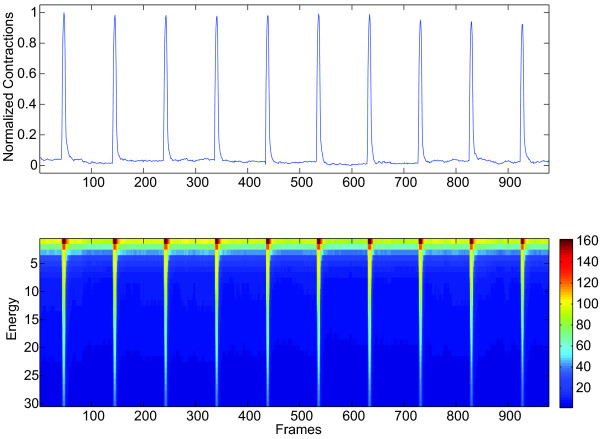
**Contractions and Energy Profile**. (a) Normalized contraction measures assessed with the proposed methodology. (b) Energy profile resulted from the deformation processes. The pseudo color intensities represent the amount of energy that is used in the warping process for each iteration and for each frame.

A typical deformation of the shape of a contracting myocyte into the shape of the average relaxed myocyte is shown in Figure 7. In this particular example, the warping process follows the path of minimum energy (as defined in the Methods section) and the warped shape matches the average shape with a correlation coefficient of over 99.99%. The perfect matching was obtained in 246 iterations after which the warping energy reached a stable minimum. This process is repeated for every frame in the video depicting the contracting myocyte and the total energy employed in each deformation is calculated. These energies represent our measure of contractility. Figure 8 shows the normalized contraction measures along with the energy profile resulted from the deformation process.

**Figure 9:**
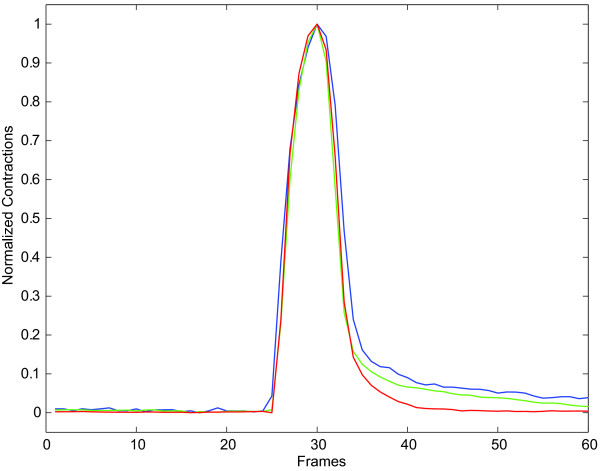
**Average Contraction**. Average contraction (10 contractions in total) as assessed by the three methods in this study: proposed method (red line), computational pipeline (green line), and raster-line (blue line).

We tested the proposed approach by assessing the contractile responses in isolated adult rat cardiocytes harvested and imaged as described in [18]. We contrasted these measurements against both the classic raster-line approach [15,11,12] and the contractility pipeline described by Bazan, Torres-Barba, Paolini and Blomgren [18]. Our results show good qualitative and quantitative agreements between the proposed method and both the raster-line method and the contractility pipeline as far as frequency, pacing, and overall behavior of the contractions are concerned. Figure 9 reproduces the average normalized contractions as assessed by the three methods in this study. We observe great similarities among the three methods, specially during the contraction or activation process marked by the electrical stimulus.

There exist small discrepancies during the relaxation phase where the raster-line method seems to show a slightly different recover process. This phenomenon was already reported in [18]. The raster-line method--being a one-dimensional technique--is unable to capture the full extent of the contraction process occurring outside its domain of influence. The proposed method, as well as the computational pipeline, capture the contraction of the cell as a two dimensional event over the entire boundary of the cell. Imaging the contracting cells with a high speed camera might eventually elucidate this small disagreement.

## Conclusions

We explored the viability of a new approach to cardiocyte contractility assessment based on biomechanical properties of the cardiac cells, energy conservation principles, and information content measures. We defined our measure of cell's contraction as being the distance between the shapes of the contracting cell, assessed by the total energy of the domain deformation (warping) of one cell shape into another. To guarantee a meaningful *vis-à-vis *correspondence between the two shapes, we employed both a data fidelity term and a regularization term. The data fidelity term is based on nonlinear features of the shapes while the regularization term enforces the compatibility between the shape deformations and that of a hyper-elastic material. Our results show good qualitative and quantitative agreements between the proposed method and both the raster-line method and the contractility pipeline as far as frequency, pacing, and overall behavior of the contractions are concerned.

We hypothesize that this methodology, once appropriately developed and customized, can provide a framework for computational cardiac cell bio-mechanics that can be used to integrate both theory and experiment. For example, besides giving a good assessment of contractile response of the cardiocyte, since the excitation process of the cell is a closed system, this methodology can be used in an attempt to infer statistically significant model parameters for the constitutive equations of the cardiocytes. This conjecture is still very preliminary. Nonetheless, the way we envision this analysis resembles that of finding the "spring constant" by measuring the deformation in the material under a constant load. In our case, we know the deformation of the cell undergoing contraction as measured by the changes in the shape. We also know that the energy that gets incorporated into the system through the electrical stimulus is the same for every contraction. For the sake of this argument, let us assume that it takes 10 snap-shots for the cell to go from its relaxed state to the fully contracted state (that is 10 shapes *S*_0_, *S*_1_, ..., *S*_8_, *S*_9_). Then, the total energy employed by the cell to go from *S*_0 _to *S*_9 _has to equal the sum of the energies for moving the cell from *S*_0 _to *S*_1 _+ *S*_1 _to *S*_2 _+ ...+ *S*_7 _to *S*_8 _+ *S*_8 _to *S*_9_. If we do this for every contraction in the experiment, we will have an over-determined system that we can solve (one of the solutions) by finding the least-square error. Note that, since we are assuming that the constitutive parameters are the same for the tissue sample, we can simply average these parameters across many cells in the same experiment.

The aforementioned possible integration between theory and experiment can also be extended further to include functional coupling between the many physiological processes that interact with mechanics such as cell growth and signaling, metabolism, transport and electrophysiology.

## Authors' contributions

CB conceived the idea and designed the methodology and the experiments; DTB conducted the biological data acquisition; PB supplied key steps for the calculus of variations--then executed by CB and verified by TH; CB and TH conducted the numerical computations; PP provided biological insight and veracity to the project; CB wrote the manuscript; All authors read and approved the final manuscript.
